# Quantitative Microscopy for Cell–Surface and Cell–Cell Interactions in Immunology

**DOI:** 10.21769/BioProtoc.5427

**Published:** 2025-09-05

**Authors:** Beatriz Díaz-Bello, Dalia El Arawi, Rémy Torro, Patrick Chames, Kheya Sengupta, Laurent Limozin

**Affiliations:** 1 Aix-Marseille University, CNRS, INSERM, LAI, Turing Center for Living Systems, Marseille, France; 2 Aix-Marseille University, CNRS, CINAM, Turing Center for Living Systems, Marseille, France; 3 Aix-Marseille University, CNRS, INSERM, IPC, CRCM, Turing Center for Living Systems, Marseille, France

**Keywords:** Cell spreading, ADCC, Image analysis software, RICM, Single-cell dynamics, Fluorescence microscopy, Time-lapse microscopy, Immunotherapy

## Abstract

Cell–surface and cell–cell interaction assays are fundamental for studying receptor–ligand interactions and characterizing cellular responses and functions. They play a critical role in diagnostics and in modulating immune system activity for therapeutic applications, notably in cancer immunotherapy. By providing time-lapsed and cell-level direct observation of the sample, optical microscopy offers strong advantages compared to current go-to techniques, which are typically either ensemble methods (e.g., measuring cell populations) or indirect readouts (e.g., impedance for adherent cells). This protocol describes two complementary microscopy-based assays: (1) a cell–surface ligand binding assay to quantify dynamic interactions between human primary Natural Killer (NK) cells and a cancer-mimicking surface, and (2) a cell–cell interaction assay to evaluate antibody-dependent cell cytotoxicity (ADCC) mediated by NK cells targeting tumor cells. Additionally, the protocol uses Celldetective, a new open graphical user interface for quantitative analysis of cell interaction dynamics from 2D time-lapse microscopy datasets. Although applied here to primary immune cells, these methods are adaptable to various cell types, including other immune cells, fibroblasts, and cancer cells. This approach enables direct observation and quantification of cellular morphology, motility, cell–cell interactions, and dynamic behaviors at single-cell resolution over time, facilitating detailed analysis of mechanisms such as cell death, migration, and immune synapse formation.

Key features

• End-to-end protocol for antibody evaluation by optical microscopy on living cells using simple reagents, followed by full open-source software image analysis and data rendering

• Quantitative analysis of cell–surface interactions using label-free imaging to study the dynamic spreading of NK cells on antibody-coated surfaces under different antibody concentrations.

• High-resolution evaluation of antibody-dependent cell cytotoxicity in NK-cancer cells co-culture using fluorescence imaging, deep learning-based death detection, and synchronized single-cell measurements.

## Graphical overview


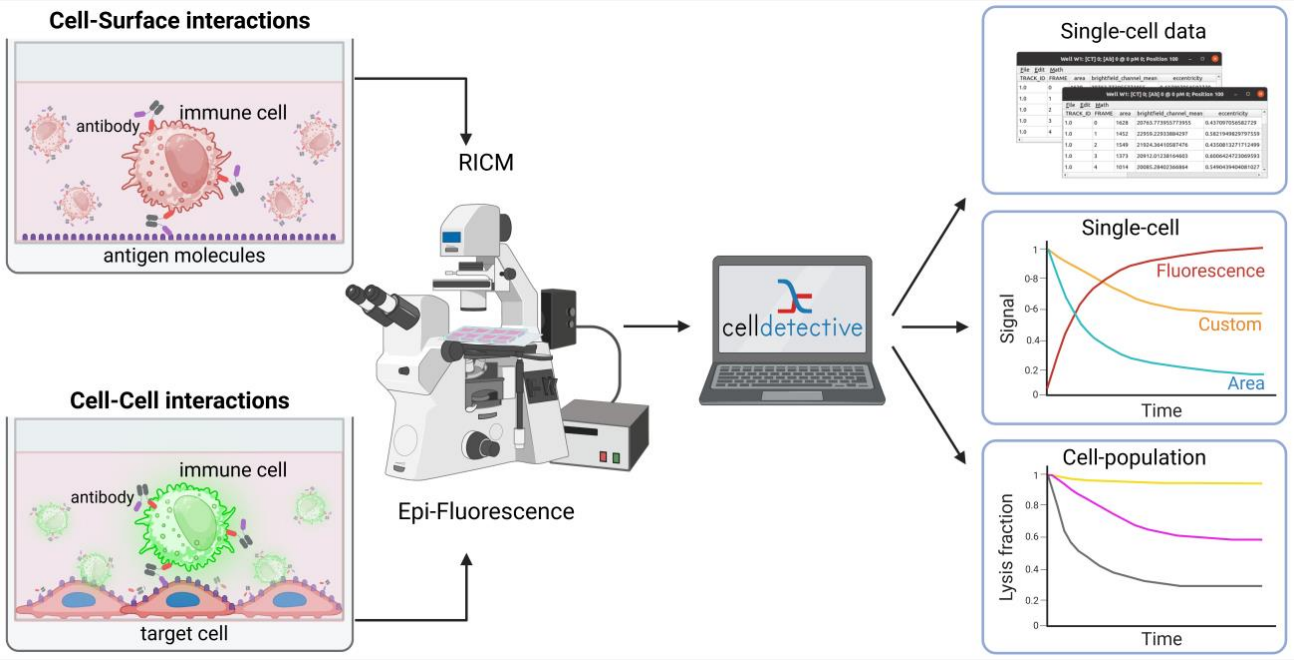


## Background

Functional immune and immunotherapy assays have become crucial for fundamental investigations as well as diagnostic and therapeutic applications. In particular, in vitro microscopy-based assays offer high control for the biological system, together with visualization capabilities and mechanistic insights. Immune cells function by forming direct contact with other cells from the immune system or with pathogenic cells, starting from activation, to shape remodeling, migration, and cytotoxicity. Therefore, a range of functional assays consists of establishing physical contact with the immune cell and monitoring its functional response [1]. Among those, there is a particular interest in the in vitro testing of potential therapeutic antibody candidates. These molecules include conventional antibody formats and bispecific ones, also called engagers [2–4], targeting NK cells or T-cells to be recruited to cancer cells. Current state-of-the-art assays rely either on time-dependent ensemble measurements (real-time cell analysis based on impedance measurements [5]) or single-cell/single pair time-independent measurements by flow cytometry [6]. None of them combine single-cell resolution and time-dependent measurements.

Cell–surface assays replace the partner cell, typically an antigen-presenting cell, with a transparent cover slide decorated with agonist molecules. This configuration enables the monitoring of cell–surface interface at high resolution, using optical surface microscopy such as total internal reflection fluorescence microscopy (TIRF [7]) and reflection interference contrast microscopy (RICM [8]). These techniques have been successfully applied to decipher fundamental mechanisms of immune cell activation and spreading [9–13]. Cell–cell assays typically involve a co-culture of immune cells with target cells [14,15]. Random or directed contacts produced by immune cell migration can lead to lysis of the target. Assessing the competition between each cell type and the outcome between proliferation and disappearance is a first step toward evaluating the efficiency of immunotherapy.

While popular, these assays are usually performed separately, with research groups rarely harnessing both to exploit their respective advantages. Also, being able to exploit these content-rich data has been largely hampered by the complexity in acquiring and analyzing microscopy sequences. Therefore, to obtain statistical significance over a large number of single-cell events remains a major challenge. Recent developments in image and sequence analysis are opening new avenues for the full exploitation of these data [16]. Taking the example of immune cell engager assessment, we provide a unified approach for harnessing these two types of assays, together with a detailed analysis protocol. As a result, we have obtained single-cell dynamics and functional information on populations of several hundred cells.

## Materials and reagents

**Biological materials**

1. Primary human NK cells (isolated from fresh blood samples of healthy donors, provided by the French Blood Establishment (EFS, Établissement Français du Sang, Marseille, France)

2. Cell line MCF7-WT (ATCC HTB-22)

3. MCF7-HER2+ cells, a genetically modified version of MCF7-WT, which overexpress HER2, described in Gonzàlez Gutierrez et al. [17]

**Reagents**

1. Fetal bovine serum (FBS) (Gibco, catalog number: 10270106)

2. Albumin, biotin-labeled bovine (Sigma-Aldrich, catalog number: A8549-10MG)

3. NeutrAvidin protein (Thermo Scientific, catalog number: 31000)

4. Biotin anti-human CD16 antibody, clone 3G8 (BioLegend, catalog number: 302004)

5. Anti-human CD3-PE antibody (Miltenyi Biotec, catalog number: 130-091-374)

6. Anti-human CD56-APC antibody (Miltenyi Biotec, catalog number: 130-113-310)

7. Anti-human CD16-FITC antibody (Miltenyi Biotec, catalog number: 130-091-244)

8. Recombinant human HER2/ERBB2 protein (His & AVI Tag), biotinylated, HPLC-verified (Sinobiological, catalog number: 10004-H27H-B)

9. Bovine serum albumin (BSA) (Sigma-Aldrich, catalog number: A9418-100G)

10. Propidium iodide (PI) (Sigma-Aldrich, catalog number: P4864-10ML)

11. Hoechst 33342 (Invitrogen, catalog number: H3570)

12. CellTrace^TM^ CFSE (Invitrogen, catalog number: C34554)

13. Bispecific antibodies (bsAb) antiCD16xHER2 (as described in Gonzàlez-Gutierrez et al. [17]) or by default, trastuzumab for research (or biosimilar like Sydlab^TM^, catalog number: C009P)

14. Dulbecco’s phosphate buffered saline (DPBS)-/-, 1× (Gibco, catalog number: 14190094)

15. RPMI 1640 medium (Gibco, catalog number: 11875093)

16. GlutaMAX^TM^ 100× (Gibco, catalog number: 35050061)

17. Trypsin-EDTA, 0.25% (Gibco, catalog number: 25200056)

**Solutions**

1. Biotin-BSA stock solution (see Recipes)

2. Biotin-BSA working solution (see Recipes)

3. NeutrAvidin stock solution (see Recipes)

4. NeutrAvidin working solution (see Recipes)

5. BSA 0.2% solution (see Recipes)

6. Anti-CD16 solution (see Recipes)

7. HER2 stock solution (see Recipes)

8. HER2 working solution (see Recipes)

9. RPMI supplemented medium for cell culture (see Recipes)

**Recipes**

**1. Biotin-BSA stock solution**

**Table:** 

Reagent	Final concentration	Quantity or Volume
Albumin, biotin-labeled bovine (4 °C)	1,000 μg/mL	10 mg
Phosphate-buffered saline, sterile	1×	10 mL

Filter with a 0.2 μm filter, aliquot in 200 μL, and store at -20 °C.

**2. Biotin-BSA working solution**

**Table:** 

Reagent	Concentration	Quantity or Volume
Biotin-BSA stock solution	1,000 μg/mL	180 μL
Phosphate-buffered saline sterile	1×	1,620 μL
Total	n/a	1,800 μL

Prepare fresh.

**3. NeutrAvidin stock solution**

**Table:** 

Reagent	Final concentration	Quantity or Volume
NeutrAvidin protein (4 °C)	1,000 μg/mL	10 mg
Phosphate-buffered saline sterile	1×	10 mL

Filter with a 0.2 μm filter, aliquot in 20 μL, and store at (-20 °C, 0.2 μm filtered, 200 μL aliquot).

**4. NeutrAvidin working solution**

**Table:** 

Reagent	Concentration	Quantity or Volume
NeutrAvidin stock solution	1,000 μg/mL	20 μL
Phosphate-buffered saline sterile	1×	1,980 μL
Total	n/a	2,000 μL

Prepare fresh.

**5. BSA 0.2% solution**

**Table:** 

Reagent	Final concentration	Quantity or Volume
BSA (4 °C)	2,000 μg/mL	0.1 g
Phosphate-buffered saline sterile	1×	50 mL

Filter with a 0.2 μm filter, aliquot in 2 mL, and store at (-20 °C, 0.2 μm filtered, 200 μL aliquot).

**6. Anti-CD16 solution**

**Table:** 

Reagent	Final concentration	Quantity or Volume
Biotin anti-human CD16 antibody, clone 3G8 (4 °C)	1.67 µg/mL	1 μL
BSA 0.2% solution	2,000 μg/mL	299 μL
Total	n/a	300 μL

Prepare fresh.

**7. HER2 stock solution**

**Table:** 

Reagent	Final concentration	Quantity or Volume
Recombinant human HER2/ERBB2 protein (-20 °C)	3,440 nM/mL	20 μg
Phosphate-buffered saline sterile	1×	65.9 μL

Aliquot in 5 µL and store at -20 °C.

**8. HER2 working solution**

**Table:** 

Reagent	Concentration	Quantity or Volume
HER2 stock solution	3,440 nM/mL	5 μL
BSA 0.2% solution	2,000 μg/mL	1,715 μL
Total	n/a	1,720 μL

Prepare fresh.

**9. RPMI supplemented medium for cell culture**

**Table:** 

Reagent	Final concentration	Quantity or Volume
RPMI 1640 medium	n/a	445 mL
FBS	10%	50 mL
GlutaMAX^TM^ 100×	1%	5 mL
Total	n/a	500 mL

Filter with a 0.22 μm filter and store at 4 °C.

**Laboratory supplies**

1. Ibidi μ-slide 8-well uncoated polymer coverslip bottom (Ibidi cells in focus, catalog number: 80821)

2. Ibidi μ-slide 8-well ibiTreat polymer coverslip bottom (Ibidi cells in focus, catalog number: 80806)

3. Cell culture treated flask T25 with vent cap (Sarstedt AG & Co., catalog number: 83.3910.002)

4. Conical tubes [Falcon, catalog number: 352096 (15 mL), 352070 (50 mL)]

5. Microcentrifuge tubes [Sarstedt AG & Co., catalog number: 72.704 (0.5 mL), 72.706 (1.5 mL), 72.695.500 (2 mL)]

6. Low protein binding microcentrifuge tubes (Eppendorf, catalog number: 022431064)

7. Filtered micropipette tips [Thermo Fisher Scientific, catalog numbers: 11752584 (10 μL), 10731194 (20 μL), 16641953 (100 μL), 11782584 (200 μL), 11749855 (1,000 μL)]

8. Serological pipettes [Falcon, catalog numbers: 357507 (2 mL), 357543 (5 mL), 86.1254.001 (10 mL), 86.1685.001 (25 mL)]

9. Immersol autofluorescence-free immersion oil 518 F (Zeiss, catalog number: 444960-0000-000)

10. Cell counting chamber slides (NanoEntek, catalog number: DHC-M01)

11. MACSxpress Whole Blood NK Cell Isolation kit (Milteny Biotec, catalog number: 130-127-695)

12. Flow filter unit 0.2 mm (Thermo Scientific Nalgene^TM^, catalog number: 566-0020)

## Equipment

1. Centrifuge (Eppendorf, Thermo Fisher Scientific, catalog number: 5805 000.010)

2. CO_2_ incubator (Thermo Scientific, catalog number: 51023121)

3. 4 °C refrigerator (Liebherr)

4. -20 °C freezer (Liebherr)

5. Single-channel micropipette, 0.2–2 μL, 2–20 μL, 20–200 μL, 100–1,000 μL (Thermo Scientific, catalog numbers: 4641010N, 4641060N, 4641080N, 4641100N)

6. Cole-Parmer^TM^ SH-200 Series See-Saw Rocking Shakers (Fisher Scientific, catalog number: 17721288)

7. MACSxpress^®^ separator (Miltenyi Biotec, catalog number: 130-098-308)

8. MACSmix^TM^ tube rotator (Miltenyi Biotec, catalog number: 130-090-753)

**Live RICM imaging**

1. Axio Observer.D1 inverted microscope (Zeiss, catalog number: 431006) (RICM) with thermostated chamber at 37 °C

2. Light source Prior lumen 200 with green filter (λ = 546 nm)

3. Camera 14-bit CCD detector (Andor iXonEM, Oxford Instruments)

4. Motorized microscope stage (Physik Instrumente, Germany)

5. Reflector module for polarizing Pol P&C (Zeiss, catalog number: 1046-279)

6. Objective Neofluar Antiflex 63× NA 1.25 oil-immersion (Zeiss, catalog number: 420489-9900) with a quarter-wave plate optimized for 546 nm; by default, a plan-apochromat 63×, NA ≥ 1.25 oil immersion objective

**Live fluorescence imaging**

1. Axio Observer.Z1 inverted microscope (Zeiss, catalog number: 431007-9901-000) with thermostated chamber at 37 °C

2. LED light source (CoolLED, pE-400max, catalog number: pE-400-MX-L-SB-SYS-ZZ)

3. Camera ORCA^®^-Flash4.0 LT+ (Hamamatsu, catalog number: C11440-42U30)

4. Motorized microscope stage (Märzhäuser Wetzlar, 2-Axes Joystick, catalog number: 00-76-200-0820)

5. Filter set cubes [Zeiss, no. 20 HE (489020-0000-000), no. 38 (000000-1031-346), no. 49 (488049-9901-000)]

6. Objective plan-apochromat 20×, NA 0.8 (Zeiss, catalog number: 420650-9903-000)

7. Objective plan-apochromat 40×, NA 1.3 oil-immersion DIC (Zeiss, catalog number: 420762-9800-000)

## Software and datasets

1. FIJI/IMAGEJ software (v. 1.54f)

2. Acquisition software: Micromanager (v. 1.4.22)

3. Analysis software Celldetective (v. 1.4.0):

• Code open source on GitHub: https://github.com/celldetective/celldetective. Sample data from the described applications available on Zenodo: https://zenodo.org/records/10650279

• Installation tutorial: https://celldetective.readthedocs.io/en/latest/get-started.html (accessed 17/07/2525)

## Procedure

**Part I: Cell–surface assay for studying NK cell spreading using RICM**

**Figure 1.**
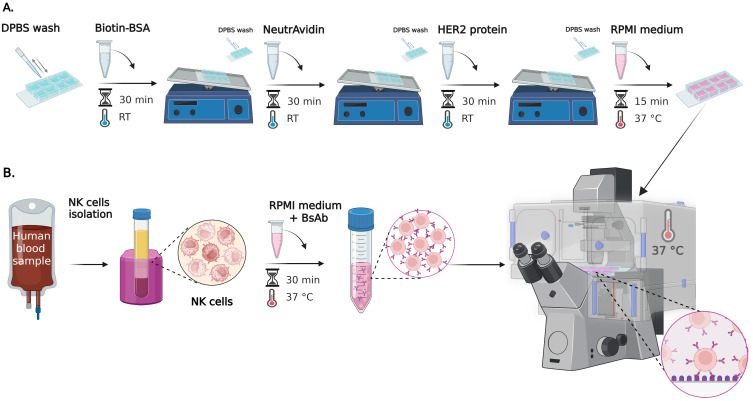
Step-by-step preparation of cell–surface assay for studying NK cell spreading using reflection interference contrast microscopy (RICM). (A) Surface preparation of 8-well chambered slides: wells are sequentially coated with biotin-BSA, NeutrAvidin, and either anti-CD16 or HER2 protein (for experimental and control conditions). Protein incubations are performed on a see-saw rocking shaker. Each coating step is followed by DPBS rinses. Positive and negative controls are included to assess NK cell activity as well as nonspecific binding to the surface. After coating, wells are filled with RPMI supplemented medium and incubated for 15 min at 37 °C. (B) NK cell preparation and live-cell imaging: primary human NK cells are isolated and characterized by flow cytometry as described in Gonzàlez et al. [12]. Cells are pre-incubated with bispecific antibodies (bsAb) (concentration 0.14–14,000 pM) before seeding onto HER2. BsAb is not added to the control well with an anti-CD16-coated surface. Live-cell RICM imaging is performed immediately after cell addition at 37 °C, capturing time-lapse sequences from multiple positions over a 10-min period.

**A. Surface preparation (Figure 1A)**

1. Rinse each well of an 8-well chambered uncoated slide with 200 μL of sterile DPBS at room temperature (RT).

2. Add 200 μL of biotin-BSA working solution to each well. Incubate for 30 min at RT on a see-saw rocking shaker set to 20 oscillations per minute.

**Caution:** Prepare BSA-biotin working solution fresh by following Recipe 2.

**Critical:** Ensure full well coverage to enable uniform protein adsorption.

3. Pipette out BSA-biotin working solution and rinse each well four times using 200 μL of sterile DPBS at RT for each rinse.

**Critical:** Avoid touching or scratching the bottom of the well with the pipette tip to preserve the homogeneity of the coated surface.

4. Add 200 μL of 10 μg/mL NeutrAvidin working solution to each well. Incubate for 30 min at RT on a see-saw rocking shaker at 20 oscillations per minute.

**Caution:** Prepare NeutrAvidin working solution fresh by following Recipe 4.

**Critical:** Ensure full substrate coverage with liquid to enable uniform protein adsorption.

5. Remove NeutrAvidin working solution and rinse each well four times using 200 μL of sterile DPBS at RT for each rinse.

**Critical:** Avoid touching or scratching the bottom of the well with the pipette tip to preserve the homogeneity of the coated surface.

6. Plan the layout of the 8-well chamber slide before beginning the coating steps. Choose one well as a positive control by adding anti-CD16 after NeutrAvidin incubation. Use the remaining seven wells for HER2 coating: one of these will serve as a negative control for NK cell nonspecific binding to the surface (no antibody), and the other six will later receive different concentrations of bispecific antibodies.

7. Add 200 μL of anti-CD16 solution (see Recipe 6) to the designated positive control well. Add 200 μL of HER2 working solution (see Recipe 8) to the remaining seven wells. Incubate for 30 min at RT on a see-saw rocking shaker at 20 oscillations per minute.

**Caution:** Prepare anti-CD16 and HER2 working solutions following Recipes 6 and 8 immediately before use.

**Critical:** Antibody solutions must be freshly prepared to maintain protein stability and coating efficiency.

*Note: Ensure consistent well labeling to distinguish control and experimental conditions.*

8. After incubation, carefully remove the solutions and rinse each well four times using 200 μL of sterile DPBS for each rinse.

**Critical:** Avoid touching or scratching the bottom of the well with the pipette tip to preserve the homogeneity of the coated surface. Always change tips between the anti-CD16 well and HER2-coated wells to prevent cross-contamination.

**B. NK cell preparation and seeding (Figure 1B)**

1. Isolate primary human NK cells from 30 mL of concentrated suspension of blood platelets (a residual blood product from whole blood donation) using the MACSxpress Human NK Cell Isolation kit. Characterize by flow cytometry with anti-CD16, anti-CD3, and anti-CD56 antibodies as described in González et al. [12].

**Critical:** Use the cells within 24 h. A prolonged culture leads to the loss of surface protein expression, critical for functional assays.

**Caution:** Human blood and blood-contaminated materials must be treated as potentially infectious regardless of the donor's health status. Thus, lab coats or gowns, gloves, and eye protection must be used when handling blood or related samples. Perform all procedures likely to generate aerosols or droplets (e.g., vortexing, centrifuging, pipetting) within a certified Class II Biological Safety Cabinet (BSC) and decontaminate work surfaces, equipment, and reusable tools at regular intervals and after completing procedures or any spill.

*Note: β-mercaptoethanol (BME) is not a standard supplement for NK cell cultures. Unlike T and B lymphocytes, where BME plays a well-established and essential role, NK cells do not require BME due to differences in their metabolic and redox needs. Established protocols for human NK cell expansion and functional assays typically rely on cytokines such as IL-2 and IL-15, along with serum or serum substitutes, without the need for BME supplementation.*

2. Resuspend NK cells at 2 × 10^5^ cells/mL in complete RPMI 1640 supplemented medium (see Recipe 9).

**Caution:** Maintain this cell concentration to ensure sufficient spacing between individual cells. Higher densities may lead to clustering, which compromises accurate single-cell analysis during imaging and downstream quantification.

*Note: A cell strainer step to remove clumps is not necessary during natural killer cell isolation from peripheral blood. Strainers are typically recommended for enzyme-digested tissues (e.g., spleen, lymph nodes, tumors), where residual debris and aggregates are common. In fresh blood samples, visible clumps are rare, especially if the samples have not been stored for extended periods or subject to freeze-thaw cycles. Additionally, NK cells are relatively fragile compared to other leukocytes, and extra mechanical processing—such as forcing cells through strainers—can increase cell stress and reduce viability. Therefore, standard NK cell isolation protocols emphasize minimizing manipulations to preserve cell viability and function.*

3. Incubate NK cells with the bispecific antibody at the desired concentration (here, we used 0.14–14,000 pM) in complete RPMI 1640 medium supplemented medium for 30 min at 37 °C with 5% CO_2_.

**Caution:** Dilute bsAb from the stock solution in BSA 0.2% solution immediately before use to maintain its efficiency.

**Critical:** Pre-incubation with bsAb ensures receptor engagement before imaging.

*Note: Optimal antibody concentration may vary depending on the specific bispecific antibody used. Perform a preliminary titration experiment to identify the concentration at which 100% of NK cells exhibit spreading. Use this to define an appropriate working concentration range for your assay. A typical titration range spans 0.001–1,000 ng/mL. For 8-well setups, prepare serial log- or half-log dilutions across wells (e.g., 0.001, 0.01, 0.1, 1, 10, 100, 500, and 1,000 ng/mL), adjusted according to the antibody’s sensitivity.*

4. Add 200 μL of RPMI supplemented medium to each well and transfer the Ibidi chamber slide to a microscope stage preheated to 37 °C. Maintain temperature throughout imaging.

5. Remove the RPMI supplemented medium and carefully add 200 μL of pre-incubated NK cell suspension to each HER2-coated well. For the positive control well (anti-CD16-coated), add 200 μL of NK cell suspension without bispecific antibody pre-incubation.

**Caution:** Avoid introducing air bubbles during pipetting, as they can interfere with cell adhesion and imaging.

**C. Live RICM imaging**

1. Start imaging immediately after NK cell addition. Acquire time-lapse images every 15–20 s per field of view and cycling through 16 positions per well over a total period of 10 min.

2. Use a 63× oil-immersion objective with high numerical aperture (NA ≥ 1.25) to ensure sufficient resolution for detecting membrane spreading and contact dynamics.

**Caution:** Ensure proper use of immersion oil—avoid air bubbles and apply neither too much nor too little—to maintain optimal image quality.

**Critical:** Carefully adjust the focus on the surface using the field diaphragm, with its opening adjusted in order to have the diaphragm visible in the corners of the image.

*Note: Image one well at a time to ensure accurate capture of spreading kinetics and contact dynamics within the defined time window.*

3. Set up Köhler illumination before imaging to optimize the contrast and ensure uniform illumination across the field.

4. Minimize exposure time while preserving contrast (e.g., ~100 ms), especially when using transmitted light or low-intensity fluorescence.

5. Maintain the sample at 37 °C using a stage-top incubator or enclosed live-cell imaging chamber. CO_2_ is not typically necessary for short-term imaging (10 min), but include 5% CO_2_ if imaging extends beyond 30 min or media buffering.

**Caution:** Prevent drift by prewarming the microscope components and sample and by ensuring stage and environmental stability throughout the acquisition.

**D. Notes and recommendations**

1. Ensure that the microscope is maintained at a stable temperature (37 °C) to ensure optimal imaging performance.

2. Clean the objective lens before imaging to remove dust or oil residues that could degrade image quality.

3. Avoid mechanical vibrations near the microscope during imaging to prevent drift or loss of focus in high-resolution acquisitions.

4. Prewarm the complete RPMI 1640 medium to 37 °C before use to avoid thermal shock to cells, which can affect their adhesion and behavior.

5. Never allow the surface of Ibidi wells to dry out; always maintain an appropriate volume of solution to preserve the integrity of the surface coating.

6. Always use freshly prepared solutions and avoid freeze-thaw cycles of proteins or antibodies to preserve their functional integrity.

7. Label all wells clearly and consistently using a permanent marker on the Ibidi slide (e.g., on the plastic frame) to prevent misidentification of control and experimental conditions during imaging and data analysis.

8. Perform a pilot experiment to optimize key parameters, such as antibody concentration, imaging interval, and focus stability, before committing to a full dataset.

9. Use the same imaging settings (exposure, gain, illumination intensity) across all wells and conditions to ensure accurate and quantitative comparison.

10. Maintain consistent incubation conditions and synchronized imaging timing across all wells to ensure reliability and reproducibility of experimental results.

**Part II: Cell–cell assay to study antibody-dependent cell-mediated cytotoxicity**

**Figure 2.**
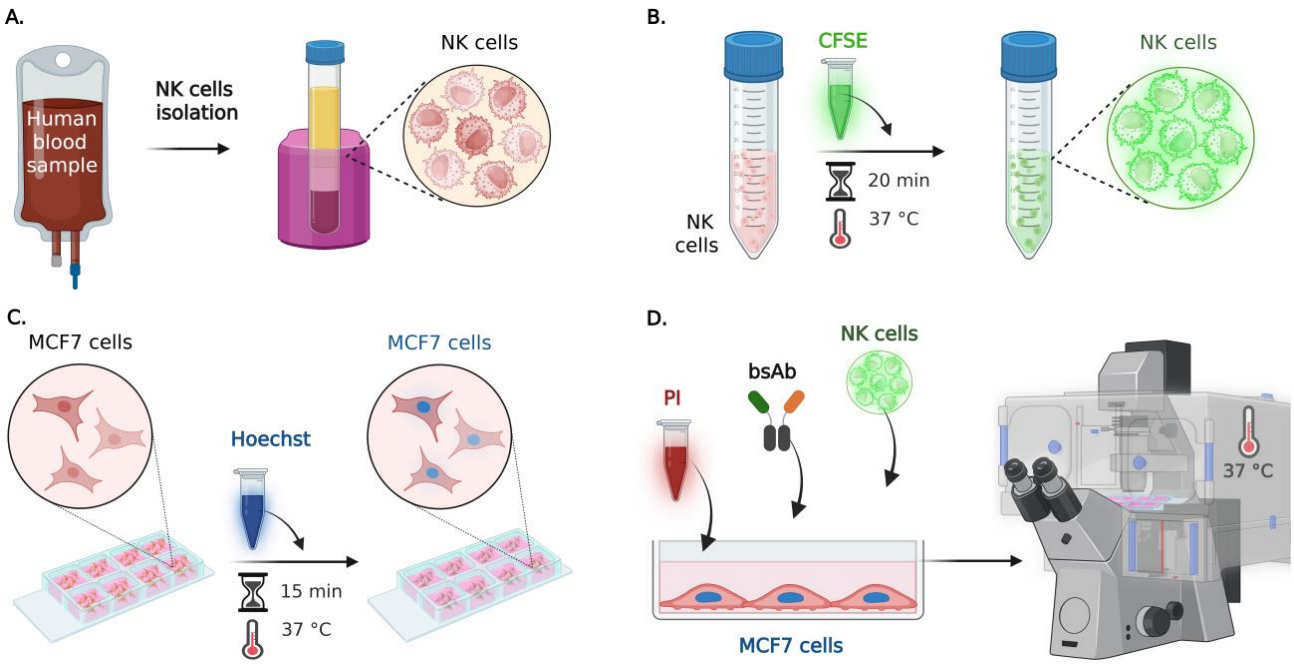
Step-by-step preparation of cell–cell assay by live fluorescence microscopy. (A) NK cells purification: primary human NK cells are isolated from whole blood and characterized by flow cytometry as described in Part I, Section B and in González et al. [12]. (B) Effector cells preparation: NK cells are incubated with the CFSE cytoplasmic green fluorescent marker for 20 min at 37 °C, before adding them to the target cells. (C) Target cells preparation: the nuclei of live MCF7-*WT* and MCF7-HER2+ cells (cultured overnight in a multiwell slide) are stained with Hoechst 33342 for 15 min at 37 °C. (D) Culture media containing propidium iodide (PI) and the selected bispecific antibody (bsAb) is added to the multiwell with the fluorescent target cells; then, fluorescent NK cells are also added to the selected wells, and live epifluorescence imaging is performed at 37 °C, capturing time-lapse sequences from multiple positions.

**A. Human primary NK cell isolation (Figure 2A)**

1. Isolate the NK cells as described in Part I, Section B.

**Critical:** Utilize the NK primary cells maximally in the next 24 h after isolation.

**B. Effector cells fluorescent staining (Figure 2B)**

1. Take the corresponding number of isolated human primary NK cells according to the desired ratio of effector to target cells (E:T) and put them in a conical tube (e.g., E:T ratio from 1:1 to 10:1).

2. Centrifuge the NK cells at 600× *g* for 5 min at RT.

3. Aspirate the supernatant and resuspend the pellet in a dilution of the green fluorescent cytoplasmic marker CFSE in DPBS. For each million NK cells, utilize 0.2 μL of CFSE diluted in 1 mL of DPBS.

**Caution:** Prewarm the DPBS at 37 °C in order to avoid using it at 4 °C directly on the cells.

**Critical:** After this point, it is important to limit the exposure of cells to light to better preserve fluorescence. If possible, avoid the light from the culture chamber.

*Note: As an optional step, the conical tube can be covered with aluminum foil.*

4. Put back the conical tube with the cells and the CFSE dilution in the incubator for 20 min at 37 °C.

*Note: The lid of the conical tube can be left slightly open during this incubation.*

5. Add 5 times more volume of supplemented RPMI to the cells (e.g., for each milliliter of CFSE dilution, add 5 mL of supplemented RPMI media, for a 6 mL final volume in total).

*Note: Consider this final volume before the incubation of the cells with the CFSE solution to not exceed the maximum capacity of the tube.*

6. Mix the cells gently with the supplemented RPMI medium by repetitive pipetting and incubate them for 5 additional minutes in the incubator at 37 °C.

7. Centrifuge the cells at 600× *g* for 5 min.

8. Aspirate the supernatant and resuspend the pellet with supplemented RPMI media according to the desired final cell concentration.

9. Put back the cells in the incubator and protect from light until use.

**Critical:** Limit the exposure of cells to light to better preserve fluorescence.

*Note: As an optional step, the conical tube can be covered with aluminum foil.*

**C. Target cell preparation (Figure 2C)**

1. Seed the target cells to obtain the desired confluence. For an 8-multiwell slide, seed between 60,000 and 80,000 MCF7-WT or MCF7-HER2+ cells per well with 300 μL of supplemented RPMI media and culture them overnight (18 h) in standard cell culture conditions of 37 °C, 5% CO_2_, and humidity of 85%–95%.

**Caution:** To evaluate precisely the number of cells to seed, assess the confluence of the target cells in a preliminary experiment. Achieve a 70%–80% maximum confluence to ensure a single layer of target cells for optimal individual cell detection during analysis.

*Note: Slides with different well numbers or plates can be used (this protocol will refer to an 8-multiwell slide with 1 cm^2^ area for each well).*

2. The next day, aspirate the media from each well and add 200 μL/well of a dilution of Hoechst 33342 in supplemented RPMI media (5 μg/mL final concentration).

**Caution:** Gently remove the media from the wells to avoid the detachment of the target cells.

*Note: From a 10 mg/mL Hoechst stock, take 1 μL and dilute it in 2 mL of cell culture media.*

3. Put back the multiwell slide in the incubator to allow nuclei staining with Hoechst 33342 for 15 min at 37 °C.

**Critical:** After this point, it is important to limit the exposure of cells to light to better preserve fluorescence.

4. Remove the Hoechst solution from the wells and rinse the cells three times with 300 μL/well of warm RPMI supplemented medium.

**Critical:** Aspirate the media and rinse the cells one well at a time to avoid dryness and detachment of the target cells.

5. Put the cells back in the incubator until use.

*Note: Cover the slide with aluminum foil if it needs to be transported to another facility.*

**D. Live fluorescence imaging (Figure 2D)**

1. Prepare the diluted solutions of the bispecific antibodies (bsAb) at the desired concentrations in supplemented RMPI medium, considering a final volume of 300 μL/well.

2. Take the multiwell slide from the incubator and gently remove the medium from the wells.

**Critical:** Aspirate the media one well at a time to avoid dryness and detachment of the target cells.

3. Add 150 µL/well of supplemented RPMI media containing propidium iodide (PI) at 8 µg/mL, considering a final volume of 300 μL/well (final PI concentration = 4 μg/mL), e.g., for 8 μg/mL, take 10 μL of a 1 mg/mL PI stock solution and dilute it in 1.25 mL of supplemented RPMI media.

4. Add 50 µL of the corresponding dilution of the bsAb.

*Note: Usual concentrations can be between 0.1 and 1,000 pM, as described in [16,17].*

5. Place the Ibidi slide on the inverted microscope with temperature control at 37 °C. Using the Micromanager software (or other appropriate microscope driver), select the fields of interest on each well and save them.

**Caution:** Use transmitted light and brightfield to select the fields of view with similar target cell confluence; avoid fields with less than 40% of confluence, because they can accumulate more effector cells in the empty spaces.

**Critical:** Avoid fields where the target cells are growing in more than one layer. This could interfere with the proper segmentation of the target cell nuclei.

6. With the help of the UV light cube filter, verify that the nuclei of the target cells are in sharp focus for acquisition and update the position information in Micromanager software.

**Caution:** Use the minimum exposure possible per position in order to avoid bleaching the sample or perturbing the cells.

*Note: If the use of the focus stabilization system is available, use it for maintaining a good acquisition and better cell segmentation during the software analysis.*

7. Once the positions and focus are saved, add the stained NK cells at the selected ratio of effector cells:target cells (E:T).

*Note: For an E:T ratio of 5:1, if 60,000 target cells were seeded, then add 300,000 stained NK cells per well.*

8. In the Micromanager software, select four channels of acquisition (brightfield, PI, CFSE, and Hoechst) and add a Z-offset if needed.

*Note: Usually, a nucleus sharper focus of living target cells is found lower than the focus of the cell in the brightfield and the dead nucleus (PI marker). Effector cells (CFSE) are observed higher than the target nucleus.*

9. Perform image acquisition using the 20×/0.8 or the 40×/1.3 objective by alternating transmitted light illumination (brightfield) and reflected light illumination (epifluorescence) for each channel.

10. Capture 5–9 fields per well over 2–5 h with minute intervals between frames (from 3 to 20 min).

*Note: Depending on the number of fields per well and the exposure time, the frame rate could vary from 3 to 20 min.*

11. Once the experiment is finished, discard the media from the wells and the cells properly.

**E. Notes and recommendations**

1. Ensure that the microscope is maintained at a stable temperature (37 °C) to ensure optimal imaging performance.

2. Propidium iodide and Hoechst 33342 are DNA intercalator molecules that need to be managed and disposed of with care.

3. Carefully add the effector cells directly under the microscope by removing the lid of the multiwell without moving the sample. Otherwise, it may significantly alter the focus previously adjusted.

4. Avoid overexposure time of fluorescence light on the living cells during acquisition.

## Data analysis

**A. Prepare image stacks (for both applications)**

1. Transfer the image TIF files to the analysis workstation. If using Micromanager, files will be in the form of hyperstacks containing several channels and time points. One file should correspond to one position (field of view) acquired on the sample.

2. Launch Fiji software and for each TIF stack:

a. Go to *Image → Properties...* to verify that the metadata is correctly interpreted.

b. Ensure the number of channels in the field *Channels (c*) is correct. If time-lapse data is present, adjust the number of frames in the field *Frames (t*) accordingly.

c. Record the following parameters:

• Image width and height in pixels.

• Spatial calibration from pixels to micrometers.

• Number of channels.

• Channel order and content (e.g., channel 1 is RICM, channel 2 is brightfield).

3. Re-export each validated image stack as a new TIF file via *File → Save As → Tiff....*

**B. Launch Celldetective (both applications)**

1. Open a console (Terminal) on your workstation.

2. Launch Celldetective with the following command: python -m celldetective

**C. Create a new experiment project (both applications)**

**Figure 3.**
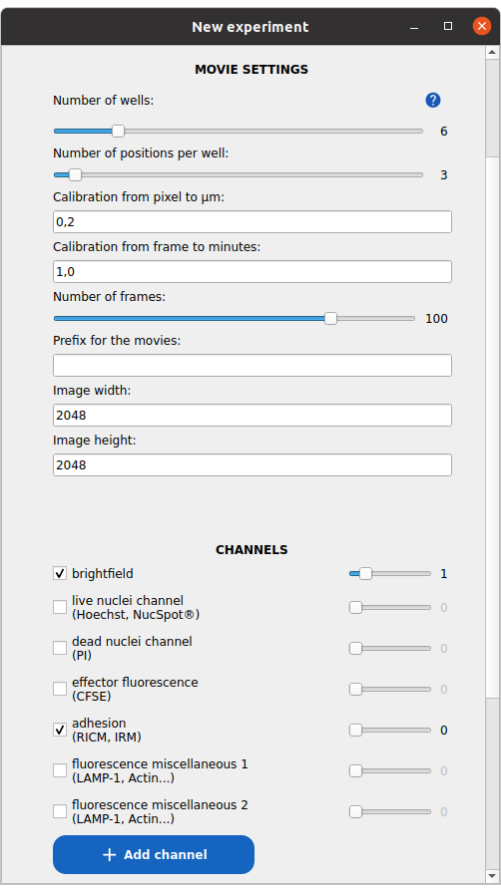
Window to set up a new experiment project. In this example, a 6-well experiment, with three fields of view in each well, is defined. The images are two-channel, with brightfield and reflection interference contrast microscopy (RICM) as the first and second channels, respectively.

In the Celldetective startup window:

1. Go to *File → New* or press *Ctrl+N* to create a new project.

2. Choose a destination folder where the project will be saved. A folder will be created to store the project.

3. In the *Experiment Name* field of the new window (see Figure 3), enter a project name. Avoid spaces and special characters. The name will be used as the folder name.

4. Specify the number of wells (i.e., experimental conditions or replicates).

5. Specify the number of positions (fields of view) per well. If the number varies between wells, enter the maximum value.

6. Enter the pixel size in micrometers (image calibration, recorded previously in Data analysis A2c).

7. Provide the average time interval between frames in minutes.

8. Enter the estimated number of time points per TIF stack. If stack lengths vary, enter the minimum.

9. Input the image width and height in pixels, recorded previously in Data analysis A2c.

10. Define the image channels:

a. Activate the relevant channels and assign the **correct index** (starting from 0).

b. Use standard naming conventions when applicable:

i. For cell–surface assays, select adhesion_channel for RICM.

ii. For cell-cell assays, select:

• brightfield_channel for brightfield.

• live_nuclei_channel for Hoechst.

• dead_nuclei_channel for PI.

• effector_fluo_channel for CFSE.

iii. To define additional channels, click on the button *Add channel*.

iv. Double-check that each channel index matches the actual channel order in your image stacks.

11. Define your cell populations:

a. Assign immune cells as effectors (for both applications).

b. Assign cancer cells as targets (for cell-cell assays only).

c. Click on the *Add cell population* button to define additional populations as needed.

**Figure 4.**
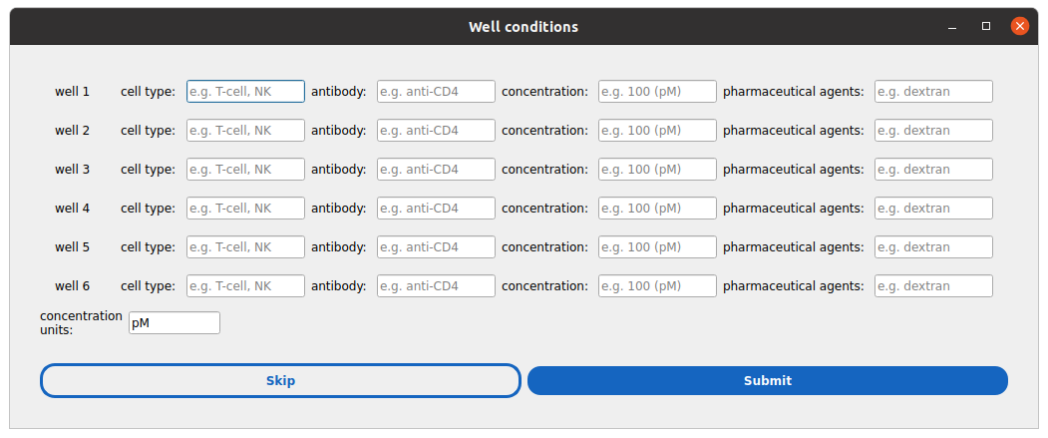
Window to define per-condition metadata. There is one row per well defined in the previous step. The basic metadata fields include the cell type, antibody, and concentration of this antibody, and the use of pharmaceutical agents. Users can expand the list of “sample” metadata later on.

12. Press *Submit* to create the project.

a. A second window will prompt sample metadata (e.g., biological conditions per well, see Figure 4). You may skip this step or fill it out and submit.

b. The project path is now registered in the Celldetective interface, and the project is loaded.

13. In the *New project* window, click the folder icon in the header (top-right) to open the project directory in your system file explorer.

**D. Organize raw image files (both applications)**

**Figure 5.**
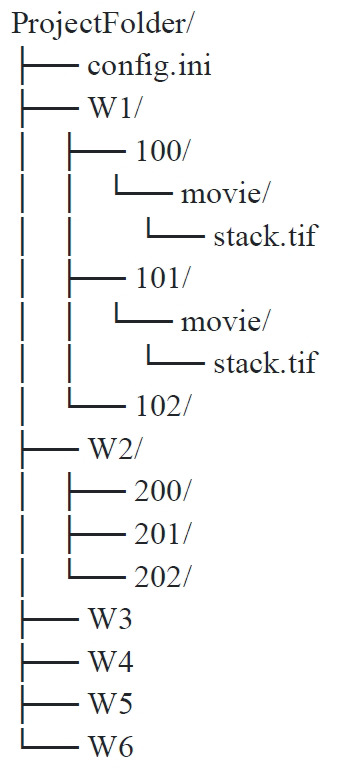
Folder architecture for dataset storage

The project folder follows this structure; here, only the first two positions of the first well are expanded to show where the TIF files have to be dropped manually (drop one TIF file per position in every project folder labeled “movie”) (Figure 5).

**E. Background normalization procedure for RICM images (cell–surface assay)**

**Figure 6.**
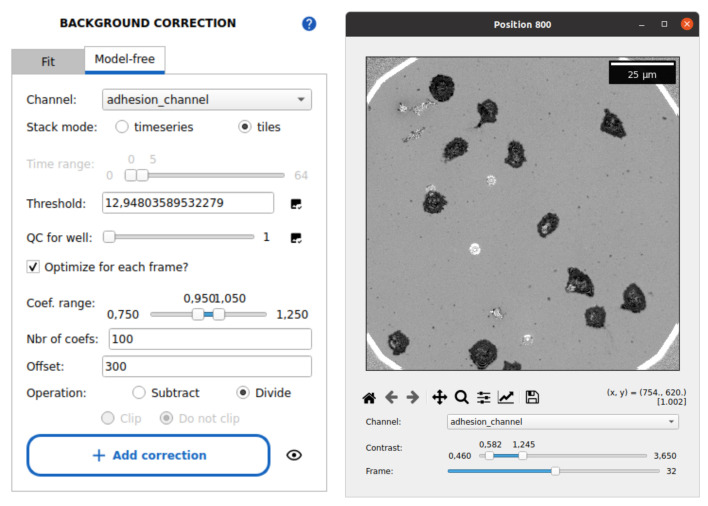
Image preprocessing with Celldetective. The model-free background correction tab is shown, with typical parameters to perform a median background reconstruction over image tiles and normalize the images. The corrected image stack is shown on the right side using the stack viewer of Celldetective (normalized RICM image of spreading primary NK cells).

**Figure 7.**
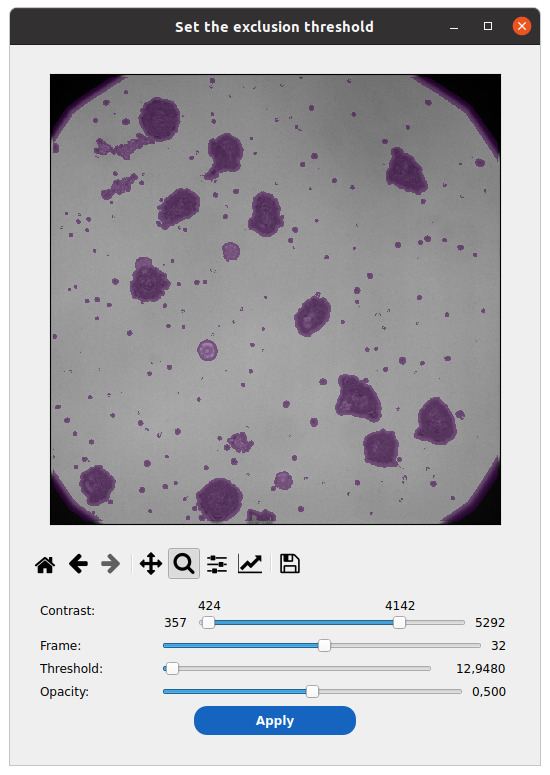
Select out the non-background part of the image. Graphically determine a threshold value on the blurred and standard deviation–filtered image to mask out cells from background estimation.

1. Open the project and expand the *PREPROCESSING* block.

2. Select the *Model-free* tab (see Figure 6).

3. Set the following parameters:

• *Channel*: Select adhesion_channel.

• *Stack mode*: select *timeseries* for time-lapse data.

• *Time range*: Adjust the slider to 0–5 frames to only include the initial frames in the background reconstruction (adapt accordingly).

4. Click on the image check icon beside the *Threshold* field to view and select the threshold that best masks cells while preserving the background signal (see Figure 7).

5. Select the option *Optimize for each frame* to adjust the background intensity for each frame. Default parameters can typically be used.

6. Set the camera black level in the *Offset* field.

7. Choose the *Divide* operation.

8. Press *Add correction* to queue the operation. Then, scroll down and click *Submit* to apply background correction across all wells and positions. Confirm the dialog prompt.

9. During processing, the GUI will freeze, and a progress bar will appear in the console.

10. After completion, click the tool icon in the header near the folder icon (top-right corner) and set the movie_prefix field to *Corrected*. This will force the software to use the background-corrected images for the analysis.

11. Use the stack viewer (button to the right of the *Position* selector, in the header) to inspect normalized images:

a. Background regions should have intensity values close to 1 and not fluctuate across frames.

b. If background correction is unsatisfactory (e.g., some frames are too bright or too dim), retry preprocessing with a broader threshold coefficient optimization range.

c. In such a case, do not forget to reset the prefix to the raw stack's original name.

**F. Single-cell analysis (both applications)**

**Figure 8.**
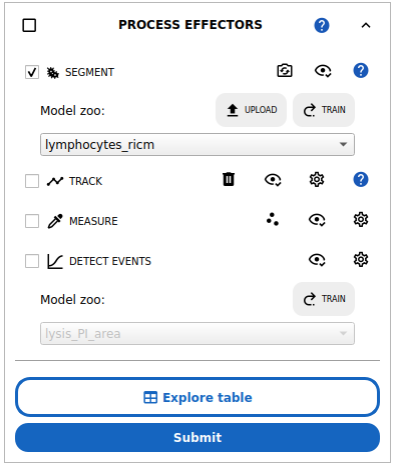
Single-cell analysis workflow in Celldetective. The workflow includes segmentation, optional tracking, measurements, and optional event detection. Here, the lymphocytes_ricm deep learning model is preselected and will be run on the selected positions upon submission.

1. Begin with a single well and position to optimize the workflow before proceeding with a large volume of data.

2. Expand the appropriate *PROCESS [POPULATION]* block to extract the single cells for the population of interest (see Figure 8).

3. Cell segmentation: select the SEGMENT option and select a segmentation model for your population.

a. For the effector cells in the cell–surface assay, with normalized RICM images, select the *lymphocytes_ricm* model in the model zoo. This model detects the interference pattern from cells close to the surface, using the normalized RICM channel only. This model was trained to ignore red blood cells by treating them as background.

b. For the effector cells in the ADCC assay, select the *primNK_multimodal* model. This model leverages several modalities to accurately segment immune cells in the cell–cell assay.

c. For target cell nuclei in the cell–cell assay, select the *mcf7_nuc_multimodal* model. This model leverages several modalities to accurately segment target cell nuclei in the presence of smaller immune cell nuclei.

**Figure 9.**
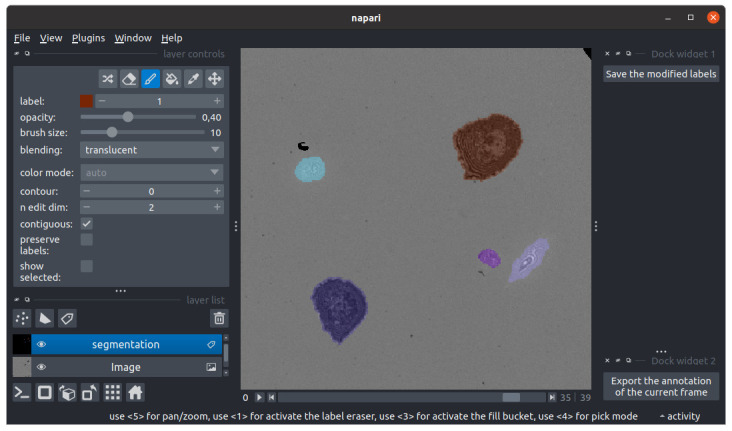
Instance segmentation masks visualization and correction in napari. Cell masks are checked in napari. Here, the segmentation layer is selected, allowing the use of the painting toolbox. The brush is selected to paint per-pixel the value 1 (corresponding to the brown spread cell). The model successfully segmented both hovering and spread cells.

4. Press the *Submit* button at the bottom of the current *PROCESS* block. A window will prompt you to select which input channel will be provided to the model, with the default being the same channels used to train the model, when available. You may also adjust the estimate for your cell size if your cells are different from the ones the model was trained on, as our models are very sensitive to cell size to be highly specific in case of population mixture. The segmentation process may take several minutes, depending on CPU or GPU speed.

5. Quality check and correction on segmentation:

a. Click the eye icon aligned horizontally with the SEGMENT option to open the masks in napari (see Figure 9).

b. Inspect visually segmentation accuracy. If the quality is good (very few mis-segmentations), you can perfect the results with manual corrections:

i. Click on the *segmentation* layer on the left side of napari.

ii. Use the pipette tool to select a cell label.

iii. Use the brush tool to add pixels; use the eraser to remove them.

iv. Press the key *M* to create a new cell label when drawing an entirely new cell.

v. Repeat for all frames as needed.

vi. Click on the *Save the modified labels* button to export corrections.

c. If the quality is too poor, consider switching to another segmentation model or a threshold-based method. For systematic errors, train or retrain a segmentation model.

6. Cell tracking: click on the settings icon aligned horizontally with the *TRACK* action.

7. Select a tracker (between *bTrack* and *TrackPy*) and tune the settings for the population of interest. Typically, set a minimum track length to 3–5 frames, and select the *Interpolate missed detection* option. Click on the save button, then select the *TRACK* option, and press *Submit* to track the population.

8. Quality check and correction on tracking:

a. Click on the eye icon aligned horizontally with TRACK to inspect tracks in napari. A process will relabel cell masks automatically with their track ID. You can then use the pipette tool of the *segmentation* layer to get the track ID from a cell.

b. If the tracks are often truncated or present a lot of errors, go back one step and change the tracking settings. If the quality is good to excellent, you can fix the remaining tracking errors:

i. Go to the last frame where the cell still has the correct track ID. Use the pipette to select it. Switch to the *Pan/zoom* tool and go to the next frame (where the cell track is wrong). Double-click on the cell with the wrong track ID. A pop-up window will ask if you want to reassign the tracking ID for this cell (and all cells from this track ID in future frames). Accept.

ii. Colors should update automatically, showing the new track ID for the cell.

iii. Click *Export the modified tracks...* to save.

9. Single-cell measurements: Click on the settings icon aligned horizontally with the *MEASURE* action. Select all of the measurements you want to perform. For both applications, there is no need to set a background correction for the measurements. Set at least in the *MASK-BASED MEASUREMENTS* section the area and intensity_mean features. In the *POSITION-BASED MEASUREMENTS*, you can set a radius of 10 pixels and the mean operation to measure intensities for all positions in the cell tracks, even if some masks are missing. Click on the *Save* button. Toggle the *MEASURE* action in the previous window and click on the *Submit* button of the *PROCESS* block. A pop-up window will show the computation progress. At the end, all the new measurements can be explored in the tables (button *Explore table*), a viewer for static measurements (the eye icon aligned with *MEASURE*), and a viewer for time-series (the eye icon aligned with *DETECT EVENTS*).

**G. Threshold-based event detection (cell–surface spreading assay)**

**Figure 10.**
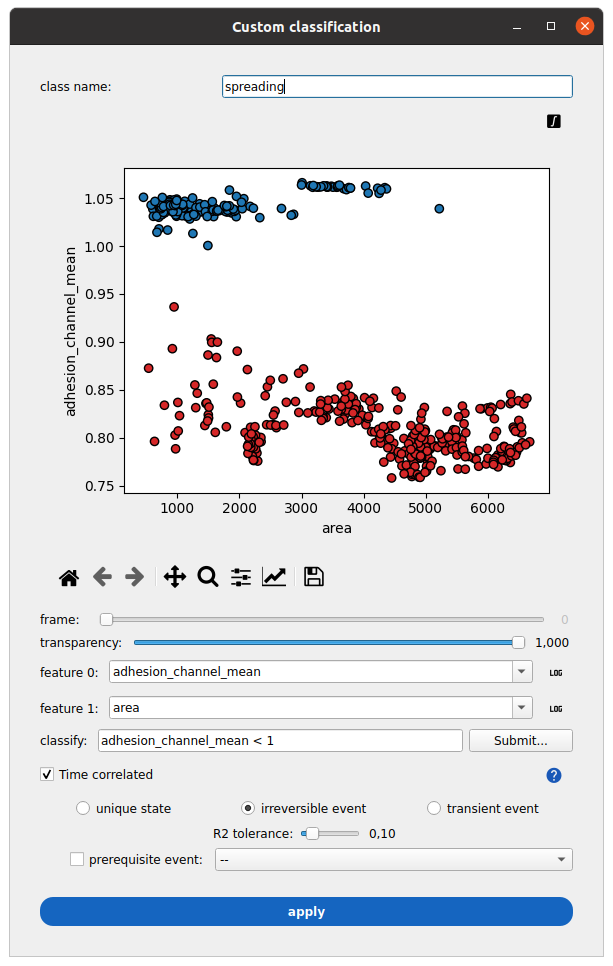
Measurements classification in Celldetective. A class name called spreading is defined. The mean normalized-reflection interference contrast microscopy (RICM) intensity over each cell mask is plotted against the mask area. In this cell–surface spreading assay example, cells are segmented from the RICM channel (adhesion_channel) only. All time point measurements are projected on the same plane. A condition separates intensity measurements larger than one (blue class) from measurements smaller than one (red class). The time-correlated option is ticked to interpret the instantaneous classification as a time series for each cell and detect irreversible spreading events.

1. Click the three-dot button aligned horizontally with MEASURE to open the classifier tool (Figure 10).

2. In the class name field, give a name to your event, such as “spreading.”

3. Set the features to be displayed on the plane (e.g., adhesion_channel_mean and area). You can press the integral sign button on the top-right corner of the plot to show the measurements for all time points on the same plane.

4. In the *classify* field, type the following condition “adhesion_channel_mean < 1” and press submit (right side) to view the result of this classification on the current plane.

5. Select the *time correlated* option.

6. Select *irreversible state* and set the R^2^ tolerance to 0.1. This will fit a sigmoid model for each cell to the binary condition at each time point. The R^2^ score is the fit quality (1 is perfect, can be arbitrarily low and negative).

7. Press the *apply* button. The window will close automatically. The event detection is represented by three values appended to each row of the single-cell table:

a. A class: Unique for each cell track regarding the event of interest (0 means an event is observed during the track, 1 that it is never observed, 2 that it happened before the beginning of the track).

b. A status: An instantaneous quantity, stating whether or not the event is “active” at this time point (1 if it is, 0 otherwise).

c. A time: For the irreversible event, this is the offset time of the sigmoid model, representing the event time when the class is 0. Otherwise, it is valued -1.

8. Click on the time series viewer. Select the event of interest (spreading). Inspect that spreading cells are properly classified (red circle) and that the event is detected at the right time (cross marker switching from blue to red, respectively, before and after spreading).

9. To correct an event:

a. Click on the cell to correct (it is selected if the marker becomes green).

b. Click on the Correct button on the left side of the viewer.

c. Select the new class (and provide the new time if the class is “event”).

d. Press the save button.

**H. Deep learning event detection (cell-cell assay)**

1. Select the *DETECT EVENT* action. Pick an appropriate model in the zoo. For cancer-cell lysis detection, you can pick the model lysis_PI_area.

2. Press the Submit button.

3. Inspect the results in the timeseries viewer (see previous section).

**I. Ensemble analysis**

**Figure 11.**
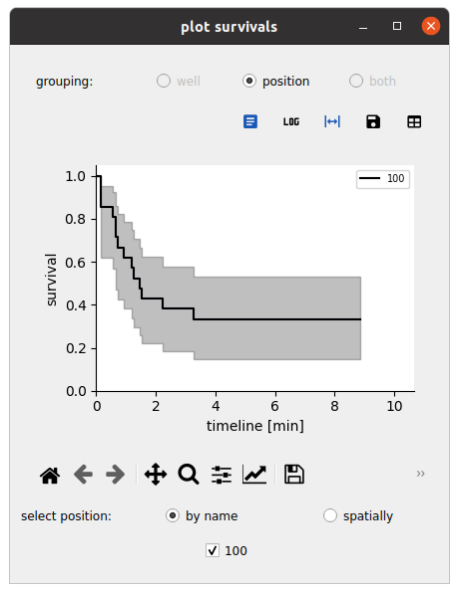
Survival representations. Exploring the survival function for an event defined by a reference and event time; here, respectively, t_firstdetection and t_spread for spreading cells. Here, the analysis is performed over a single field of view.

1. Repeat the above workflow (background correction, segmentation, tracking, measurements, event detection) for each position in each well. Use the *Select All* buttons at the top of the interface to automate the step. Perform visual quality checks for all positions.

2. Navigate to the *Analyze* tab and click on the *plot survival* button:

a. Set the population to the one of interest for the survival (effectors for the cell–surface assay, targets for the cell–cell assay).

b. Set the time of reference (t_firstdetection for the cell–surface assay, 0 for the cell–cell assay).

c. Set the time of interest for the event (t_spread for the cell–surface assay, t_lysis for the cell–cell assay).

d. Leave the *select cells with query* field blank to keep all cells.

e. Press the *Submit* button to generate survival curves across wells and conditions (see Figure 11).

**Figure 12.**
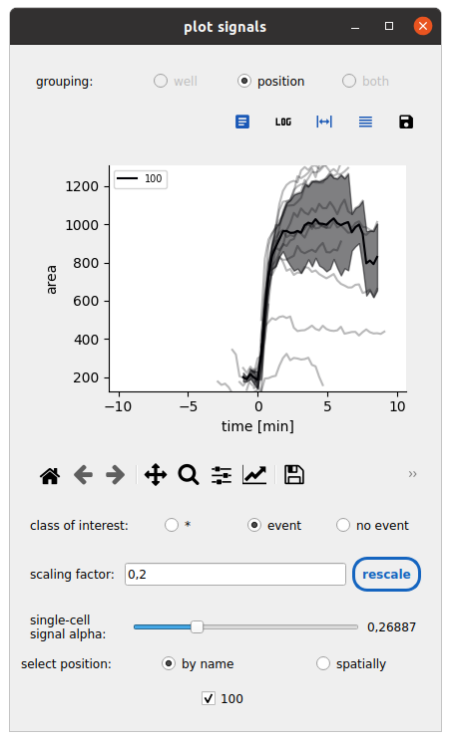
Synchronized time series representations. The mask area response at the onset of spreading is synchronized for cells in the cell–surface spreading assay. Only spreading cells are shown by setting the class of interest. The average trend and standard deviation are shown here across all cells from a single field of view.

3. Click on the *plot signals* button to synchronize single-cell time series with respect to an event time and compare conditions:

a. Set the population to the one of interest.

b. Set the class to the class of interest.

c. Set the event time to the one associated with the class of interest.

d. Set the pooling option for the ensemble time series (mean, median) and the minimum number of cells needed to compute this pooled time series.

e. Click *Submit*, select a feature, and browse the ensemble response across conditions (see Figure 12).

**J. Interaction analysis**

**Figure 13.**
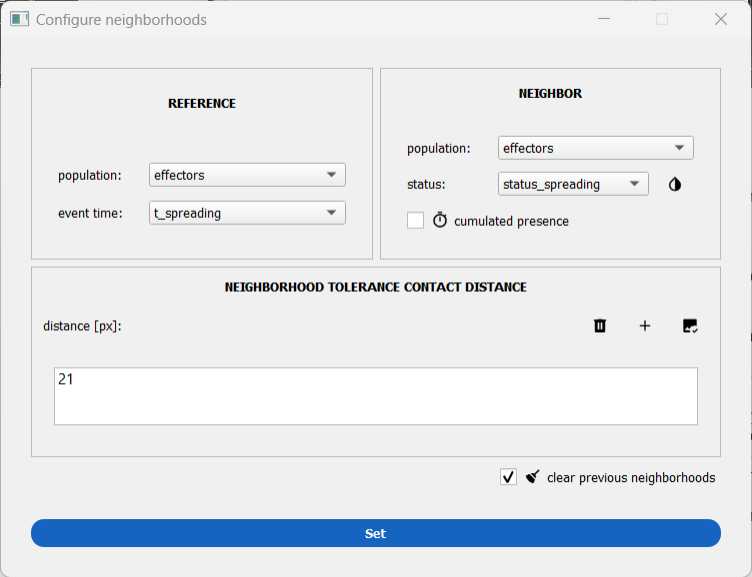
Window to set up mask-contact neighborhood. Here, the neighborhood is computed between primary NK cells in the cell–surface spreading assay that may be hovering or spread at any given time point.

**Figure 14.**
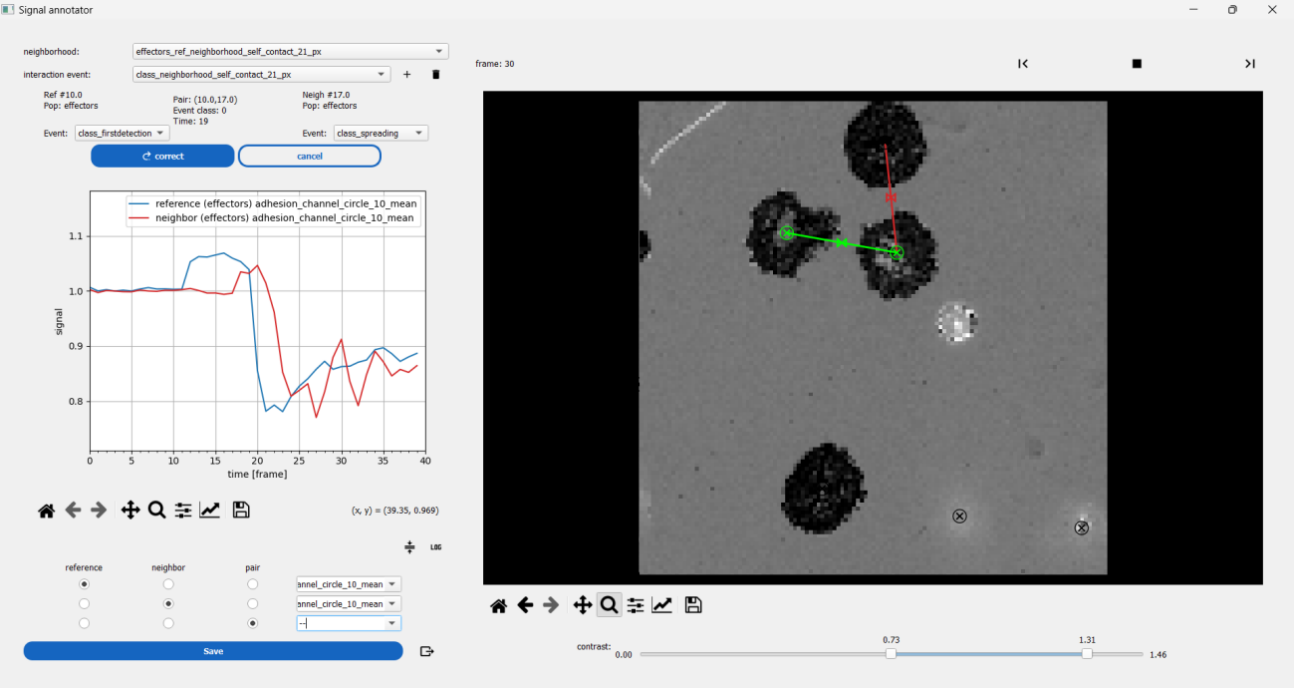
Interaction visualization. Interacting primary NK cells in the cell–surface spreading assay are explored with a dedicated viewer. The reference cell is selected first (green circle on the right side), allowing the view of all neighbor cells at each time point as moving segments. Clicking on one of the segments allows selecting a cell pair and viewing on the left side the associated measurements. Here, the blue time series is the mean normalized–reflection interference contrast microscopy (RICM) intensity for the reference cell. The red time series is the same quantity as its neighbor, showing that this specific neighbor cell spreads a few frames after the reference cell.

1. Expand the *INTERACTIONS* block.

2. Select the *NEIGHBORHOODS* action and choose a method among “isotropic distance threshold” and “mask contact.” For the cell–surface spreading assay, since complete cell masks are available, cell proximity can be evaluated with the mask contact method. In the cell–cell assay, without knowledge of the cancer cell shape in case of nuclear staining, an isotropic distance threshold is preferred to define the neighborhood.

3. Click on the plus button next to either method to set up the neighborhood computation (see Figure 13).

4. Define the reference and neighbor populations (among the ones analyzed before). For the cell–surface assay, define the effectors as both the reference and the neighbor population. In the cell–cell assay, you could define the targets as reference and the effector cells as neighbors (it is permutable).

5. (Optional) Attach an event time to the reference population. This allows the computation of the mean number of neighbors before and after the event time for the reference cells. For the cell–cell assay, this is helpful to estimate the number of effector neighbors around a target cell before the latter dies at t_lysis.

6. (Optional) Attach a status to the neighbor population. This will allow a decomposition of the neighbor counts with respect to that status (as *count_s0* for the neighbors with a status equal to 0 and *count_s1* for the neighbors with a status equal to 1). In the cell–surface spreading assay, this can be used to differentiate the number of hovering effector cells from the number of spread effector cells around a given effector cell.

7. Set the distance. Click on the image check button next to the plus button to open a viewer to tune on the image the neighborhood distance of interest (either a mask expansion with mask contact or a circle diameter with isotropic distance threshold).

8. Click on the *Set* button to validate the neighborhood distance(s).

9. In the previous window, select the MEASURE PAIRS action and press Submit to launch the computation.

10. Press the eye icon aligned horizontally with the *DETECT PAIR EVENTS* action to open the interaction viewer (see Figure 14).

11. Click on a reference cell to see its neighbors dynamically. Click on the linking segment or the neighbor cell to activate the pair. Set the time series to observe for either the reference cell, the neighbor cell, or the pair on the left side of the interface. Annotate or correct an annotation for the pair.

## Validation of protocol

This protocol has been used and validated in the following research article(s):

• Torro et al. [16]. Celldetective: an AI-enhanced image analysis tool for unraveling dynamic cell interactions. eLife14:RP105302 (doi.org/10.7554/eLife.105302.1)

## General notes and troubleshooting

**General notes**

**Constraints on the assay for analysis**

**Dimensionality:** The analysis workflow was designed for 2D + time-lapse microscopy and supports multiple fluorescence channels. It is not suited for full 3D + time acquisition. While it is technically possible to replace the time axis with a Z-axis, simultaneous analysis of Z and time dimensions is not supported. In Z-stack mode, segmentation and measurements are performed slice by slice, and tracking connects features across slices. The annotation interface loops through Z-slices, allowing users to annotate single cells within the volume.

In practice, imaging in 3D leads to lower throughput, increased phototoxicity, and greater analysis complexity. For example, increasing target cell density in the cell–cell assay leads to partial stacking of cells (a 2.5D situation), where overlapping nuclei make neighborhood analysis more ambiguous. There is already extensive biological content to explore in 2D, and adding full 3D complexity is generally not necessary for this context.

Developed for specific cell–surface and cell–cell interaction assays using primary immune cells (here, human NK cells), the workflow was already tested on human and mouse T-lymphocytes. It is, in principle, adaptable to other cell types and interaction models. The deep learning–based analysis modules provide flexibility to accommodate different dyes, imaging modalities, or assay formats. Applying the workflow to new conditions may require retraining models on representative images, particularly if cell morphology or labeling strategies differ significantly. The analysis software allows performing all the steps, from annotations to model retraining.

**Phototoxicity and dye management:** Primary immune cells are sensitive to light exposure, so it may be required to decrease light exposure in cell–cell assays to last for a few hours. Using label-free RICM avoids dyes to perturb cell response. Different dyes could be used thanks to the flexibility of deep learning–based analysis modules. In the long run (hours of acquisition), photobleaching or dye transfer between cell populations can be observed. This may require fine-tuning in the segmentation and signal analysis to account for these effects.

**Cell viability and state:** Experiments were performed using freshly isolated primary immune cells, ideally within the first 24 h after extraction from blood bags. Attempts to extend this window led to reduced viability and altered cell behavior, particularly in dynamic assays.

**Cell density:** In cell–surface spreading assays, cells should be seeded at low density to ensure that they sediment sparsely. High local density can lead to unintended cell–cell contacts, which may confound the interpretation of single-cell behaviors and reduce the reliability of surface interaction measurements. If cell–cell proximity interferes with analysis, we recommend applying filters based on local neighborhood metrics to exclude ambiguous cases. For example, the field inclusive_count_neighborhood_self_contact_25_px == 0 in the *Select cells with query* panel (see Data analysis step F2) can be used to retain only isolated cells for downstream analysis. In cytotoxicity assays or other cell–cell interaction formats, excessively high effector cell density may result in overlapping or obscuring interactions. This can make it difficult to track individual events (e.g., lysis), reduce segmentation quality, and increase analysis ambiguity. Adjust effector-to-target ratios accordingly.

**Plate format:** The current protocol is demonstrated for 8-well Ibidi slides. It has also been successfully tested with 18-well (ibidi, #81816) or 96-well plates (Nunclon^TM^ Delta Surface, #167008). However, the setting of multi-position and focus can be a time-consuming task in this case.

**Memory requirements:** To reduce memory demand, image analysis is performed at one time point at a time. Even for tracking, single-cell features are computed per-frame, and the linking across frames is performed afterward based on the much smaller tabular data, not the raw images. This significantly lowers RAM and storage requirements during processing. The main memory bottleneck occurs during visualization and manual curation. To comfortably run Celldetective, we recommend a minimum of 4 GB of RAM. The napari viewer loads the full image stack into memory for segmentation and tracking review. Users should ensure sufficient RAM to load complete image sequences during this phase (as a rule of thumb, the RAM should be twice the size of the heaviest image stack).
